# The Excellence in Translational Medicine Award 2006–07

**DOI:** 10.1186/1479-5876-5-40

**Published:** 2007-08-28

**Authors:** Richard J Ablin, Yi-Xin Zeng

**Affiliations:** 1 Department of Immunobiology, University of Arizona College of Medicine and the Arizona Cancer Center, Tucson, AZ 85724-5049, USA; 2 Sun Yat-sen University Cancer Center, Guangzhou 510060, China

## Abstract

In our endeavor to recognize outstanding contributions in the field of translational medicine, the Editorial Board of the **Journal of Translational Medicine **(JTM) in collaboration with **Global Translational Medicine at Pfizer **established "The Excellence in Translational Medicine Award" in May 2006. Fifteen nominated papers from investigators representative of eight countries covering a wide range of disciplines published in JTM between 1 July 2006 and 30 June 2007 were evaluated. The four finalists and the winner are announced in this editorial.

## Editorial

In our endeavor to recognize outstanding contributions in the field of translational medicine, the Editorial Board of the **Journal of Translational Medicine **(JTM) established "The Excellence in Translational Medicine Award" in May 2006 [1].

The recipient of "The Excellence in Translational Medicine Award" will receive a $2,500 prize sponsored by *Global Translational Medicine*, *Pfizer Global Research and Development *to cover expenses for any meeting sponsored by a non-for-profit organization that is relevant to the goal of translational medicine and research.

Fifteen nominated papers from investigators representative of eight countries covering a wide range of disciplines published in JTM between 1 July 2006 – 30 June 2007 were evaluated. For this purpose, an Award Committee* comprised of ten members of the Editorial Board selected and co-chaired by Richard J. Ablin (University of Arizona College of Medicine and the Arizona Cancer Center, Tucson, AZ) and Yi-Xin Zeng (Sun Yat-sen University Cancer Center, Guangzhou, China) was formed. The National Institutes of Health Scoring System of 1–5, with 1 = Outstanding and 5 = Poor were used with the papers being evaluated with regard to their:

• Scientific merit

• Originality

• Clarity

• Relevance to the purposes of translational medicine and research

• Research design

• Methodology

The competition, judged from the closeness of the scores, wherein 10 of the 15 papers evaluated were separated by ≤1 point and the top 4 papers by ≤0.4 points, was "fierce." The best score and winner of the "Excellence in Translational Medicine Award" was Xinmei Zhu and colleagues from the University of Pittsburgh School of Medicine; Pittsburgh Cancer Center; Geneva University and Oncovir for the paper entitled: "Toll-like receptor-3 ligand poly-ICLC promotes the efficacy of peripheral vaccinations with tumor antigen-derived peptide epitopes in murine CNS tumor models" [2].

Exemplifying the criteria for the "Excellence in Translational Medicine Award," Zhu et al. [2] proceeded from a potentially clinically relevant hypothesis through utilization of a sound research design with rigorous methodology to demonstrate with clarity that poly-ICLC (polyinosinic-polycytidylic acid stabilized with poly-lysine and carboxymethylcellulose) assisted antigen-specific peripheral vaccinations may represent an efficacious and safe therapeutic approach for central nervous system tumors.

The papers by the other three finalists: Kluger et al. [3]; Provenzano et al. [4] and Grasso et al. [5] on the subjects of the x-linked inhibitor of apoptosis protein in metastatic melanoma, characterization of the p53-binding domains of the human polyomavirus BK tumor antigen, and ejaculatory disorders and the effects of α_1_-adrenoreceptor antagonists therapy, all translational related topics followed as listed, where but separated by a hair from the winning paper by Zhu et al. [2].

With congratulations to Xinmei Zhu and colleagues [2], the *1st *"Excellence in Translational Medicine Award" is now history. We are hopeful this Award will serve to encourage other investigators devoted to improving the "bench to bedside" concept of translational medicine and initiatives in this direction. **And**, with the competition now open for the *2nd *"Excellence in Translational Medicine Award," we very much look forward to the opportunity of selecting next year's winning paper.

*"Excellence in Translational Medicine Award Committee": Richard J. Ablin (Co-Chairman); Howard L. Kaufman; Bruce Littman; Upender Manne; Pier Giorgio Natali; Hideho Okada; Michael Perricone; Raj K. Puri; Noriyuki Sato; Patrick F. Terry; Craig Webb and Yi-Xin Zeng (Co-Chairman).
